# Diagnostic Accuracy of Auricular Morphometry in Sex Estimation: A Logistic Regression Model with ROC-Based Validation

**DOI:** 10.3390/diagnostics16121820

**Published:** 2026-06-12

**Authors:** Serdar Babacan, Güven Özkaya

**Affiliations:** 1 Department of Anatomy, Faculty of Medicine, Bursa Uludağ University, 16059 Bursa, Türkiye; 2 Department of Biostatistics, Faculty of Medicine, Bursa Uludağ University, 16059 Bursa, Türkiye; guvenozkaya@gmail.com

**Keywords:** forensic anatomy, auricular morphometry, sex estimation, ROC analysis, logistic regression, forensic identification, diagnostic accuracy

## Abstract

**Background/Objectives:** Anthropometric measurements provide essential normative datasets that form the foundation for clinical practice and forensic identification. The human ear is a highly informative structure due to its complex morphology and individual specificity, making it a valuable tool for biometric systems. This study aimed to estimate biological sex based on auricular morphometric measurements, develop a logistic regression model for this purpose, and validate its performance using ROC analysis. **Materials and Methods:** This cross-sectional study included 120 adult participants (60 males, 60 females). Standardized digital photographs were analyzed in ImageJ to record 22 linear and 6 angular measurements using established anatomical landmarks. LASSO logistic regression was employed for variable selection and model shrinkage. The final model’s discriminative performance was assessed using the area under the receiver operating characteristic (ROC) curve (AUC), the Hosmer–Lemeshow test, and the Brier score. **Results:** A comparative analysis revealed that most linear and angular measurements showed significant sexual dimorphism. Almost all linear dimensions (A1–A22) were significantly larger in males (*p* < 0.001). Auricular width (A2) and width at the level of the tragus (A3) emerged as the most robust indicators, demonstrating “very large” effect sizes. Conversely, the angle between the preauricular line and the vertical plane (A28) was significantly greater in females, providing a unique inverse relationship for sex estimation. A parsimonious 5-predictor model (incorporating A2, A3, A5, A10, and A28) achieved exceptional discriminative performance with an AUC of 0.980. **Conclusions:** Auricular morphometry is a highly effective tool for sex estimation. The findings confirm significant sexual dimorphism in the external ear, particularly in linear dimensions. The developed model may serve as a preliminary morphometric reference for future automated biometric recognition studies, although no artificial intelligence-based classification model was developed in the present study.

## 1. Introduction

Anthropometry is the systematic scientific measurement of the human body and provides essential normative datasets widely used across multiple disciplines. These include clinical practice, forensic identification, ergonomics, and the design and development of biomedical devices. Through standardized human measurements, anthropometry enables objective evaluation of biological variation within and between populations [1,2].

Knowledge of human anatomy plays an important role in anthropological research and contributes significantly to sex estimation, which is a fundamental step in personal identification. In forensic anthropology, anatomical analyses provide practical and economical approaches for identifying individuals, particularly in challenging conditions such as mass disasters, burned remains, severe decomposition, or fragmented skeletal findings where conventional identification methods may not be applicable [3,4,5,6].

A central objective in forensic anthropology is the identification of individuals through the estimation of key biological parameters such as age, sex, and stature. These estimations form the foundation of the biological profile and are critical in establishing identity, particularly in cases involving unknown or decomposed human remains [7,8].

Personal identification is fundamentally based on the evaluation of morphological characteristics that exhibit individual specificity. Human populations demonstrate substantial biological variability, which provides the basis for distinguishing one individual from another with a high degree of reliability. This variability is essential for both clinical assessment and forensic identification purposes [1,9,10].

Forensic clinical anatomy applies anatomical knowledge and methods, from ultrastructural to macroscopic levels, to resolve medicolegal problems. Centered on the individuality of anatomy, it requires a detailed assessment of a person’s unique anatomical features to understand their forensic implications [11].

In addition to DNA profiling, a variety of morphological and biometric traits have been widely applied in forensic science for individual identification. These include fingerprints, facial features, footprints, and the external ear. Among these, the human ear is particularly notable due to its complex morphology and high degree of individual specificity. Similar to fingerprints, ear morphology can even be used to differentiate genetically identical individuals, including monozygotic twins, highlighting its strong potential in forensic applications [9,12,13].

Within this framework, the external ear represents a highly informative structure from both anatomical and anthropometric perspectives. Its measurable dimensions demonstrate consistent population-specific patterns, as well as significant sex- and age-related variations. These characteristics make the auricle a valuable anatomical region for both clinical assessment and forensic interpretation, particularly in studies aiming to quantify biological variation across populations [14,15].

Morphological and morphometric features of the external ear are not only relevant for maintaining facial symmetry and aesthetic proportions but also exhibit a high degree of individuality. In this regard, the ear shares key biometric properties with established identifiers such as fingerprints, the iris, and voice patterns. These unique structural characteristics support its increasing use in biometric systems and forensic identification frameworks [16,17].

The aim of this study was to estimate biological sex based on auricular morphometric measurements and to develop a logistic regression model for this purpose. The discriminative performance of the model was further evaluated using ROC analysis to determine the accuracy and reliability of ear-based parameters in sex estimation. The findings are expected to contribute to forensic identification practices and may also provide a preliminary morphometric reference for future biometric recognition and artificial intelligence-assisted classification studies.

## 2. Materials and Methods

Study Design and Sample Size Determination

This cross-sectional observational study was designed to investigate sex estimation using auricular morphometric parameters. The required sample size was determined based on previous studies in auricular morphometry and sex estimation. Considering reported moderate effect sizes in the literature, a power analysis was conducted with an alpha level of 0.05 and a statistical power of 80%, indicating that a minimum of 60 adult males and 60 adult females (total *n* = 120) would be sufficient to ensure reliable group comparisons and robust classification performance using logistic regression models.

Ethical Considerations

Ethical approval for this study was obtained from the Health Research Ethics Committee of Bursa Uludağ University prior to the commencement of data collection (protocol code: 2026/197/7-8). The study protocol, including participant recruitment, image acquisition procedures, and data handling processes, was reviewed and approved by the relevant institutional board. All procedures performed in this study involving human participants were conducted in accordance with the ethical standards of the institutional and/or national research committee and with the 1964 Declaration of Helsinki and its later amendments or comparable ethical standards. Written informed consent was obtained from all participants prior to inclusion in the study. Participants were informed about the study objectives, procedures, potential risks, and their right to withdraw at any stage without any consequences.

Participants and Sampling Strategy

Participants were recruited as adult volunteers using a non-probability convenience sampling approach. Eligible individuals who met the predefined inclusion criteria and agreed to participate were enrolled until the target sex-balanced sample size was reached, consisting of 60 males and 60 females. The recruitment process was not based on participant-driven chain referral or snowball sampling. All participants received detailed verbal and written information about the study objectives, image acquisition procedures, and data usage, and written informed consent was obtained prior to inclusion.

Inclusion Criteria for Participants

Participants were eligible for inclusion in the study if they met all predefined criteria. Only adults aged 18 years and older were recruited, and all participants were required to provide voluntary participation along with written informed consent prior to inclusion. Eligible individuals were required to have normal auricular anatomy without any congenital external ear anomalies. Furthermore, participants with a history of trauma affecting auricular morphology or any previous surgical intervention to the auricular region, including esthetic or reconstructive procedures, were excluded from eligibility. In addition, participants needed to be physically capable of maintaining the required standardized positioning during image acquisition to ensure accurate and reproducible morphometric assessment.

Exclusion Criteria for Participants

Participants were excluded from the study if any condition was present that could potentially alter the natural morphology of the auricle or compromise measurement reliability. Accordingly, individuals with congenital auricular malformations or dysmorphic features, as well as those presenting with significant structural deformities of the external ear, were excluded. A history of severe auricular trauma, including burns, lacerations, or tissue loss, also constituted an exclusion criterion. Similarly, participants with prior esthetic or reconstructive surgery involving the auricular region were not included. Additional exclusion criteria comprised the presence of infections, keloids, hematomas, or neoplastic conditions affecting the auricle, as well as piercing-related deformities or tissue alterations that could influence ear morphology. Finally, individuals unable to comply with the imaging protocol due to physical or cognitive limitations were excluded from the study.

Image Acquisition and Standardization Protocol

Auricular data were obtained through standardized digital photography. All photographs were acquired from the right side of each participant, and all auricular measurements were performed on the right auricula under standardized imaging conditions. All images were captured in a controlled environment under homogeneous lighting conditions to minimize shadowing and illumination bias. A single calibrated digital camera was used for all recordings, with fixed distance, focal length, and exposure settings to ensure measurement consistency. Participants were positioned in the natural anatomical head position, aligned with the Frankfurt horizontal plane. Lateral facial images (right side) were obtained, ensuring full visibility of the right auricle. Hair was secured away from the auricular region, and any accessories (e.g., earrings) were removed prior to imaging. A calibration scale (ruler with known dimensions) was placed within the photographic field to allow for spatial calibration during morphometric analysis. All images were acquired by a single trained investigator to reduce inter-observer variability and measurement bias.

Morphometric Measurements

**Figure 1:**
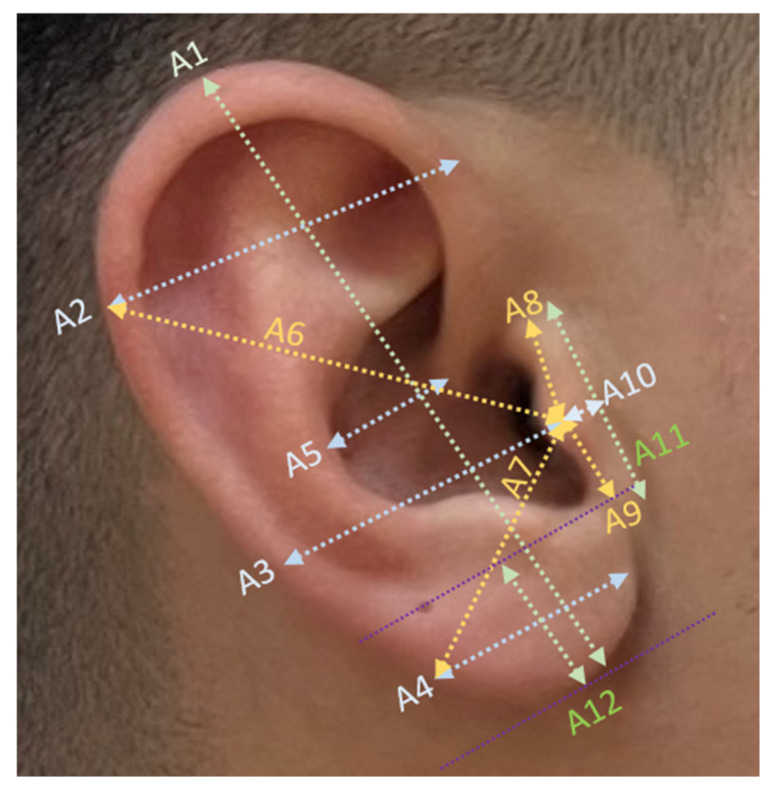
Linear measurements of the auricle (mm) (Parameters of A1–12). A1. Auricular height; A2. Auricular width; A3. Auricular width at the level of tragus; A4. Lobular width; A5. Distance between crus helicis and conchal margin; A6. Maximum distance between tragus and helix; A7. Maximum distance between tragus and lobule; A8. Upper tragal length; A9. Lower tragal length; A10. Tragal width; A11. Tragal length; A12. Lobular length.

**Figure 2:**
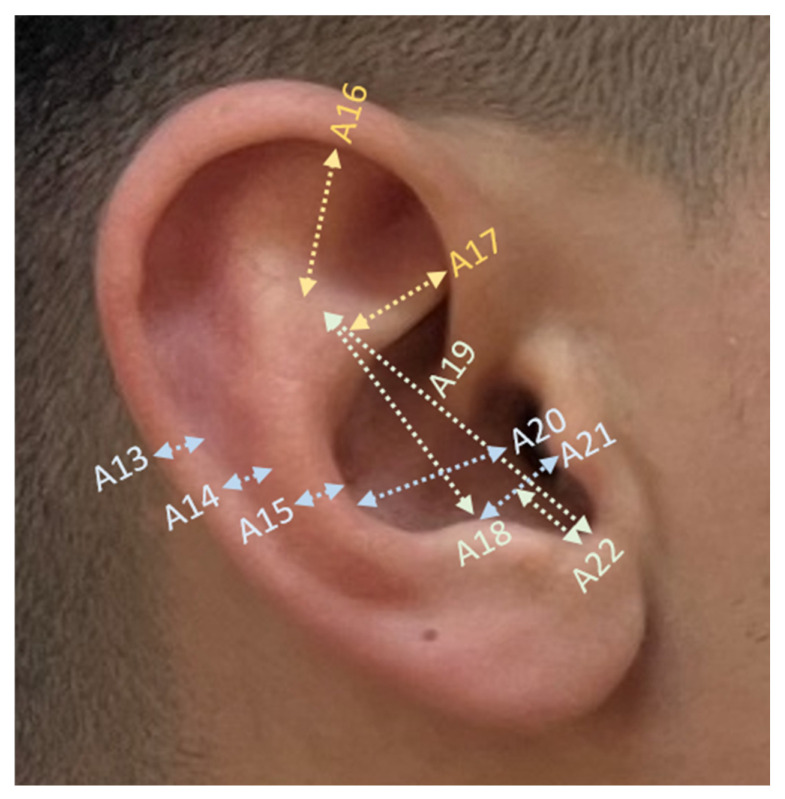
Linear measurements of the auricle (mm) (Parameters of A13–22). A13. Helical width; A14. Scapha width (helix–antihelix distance); A15. Antihelical width; A16. Superior antihelical crus length; A17. Inferior antihelical crus length; A18. Distance between crus origin and antitragus; A19. Conchal length; A20. Conchal width; A21. Tragus–antitragus distance; A22. Intertragic notch length.

**Figure 3:**
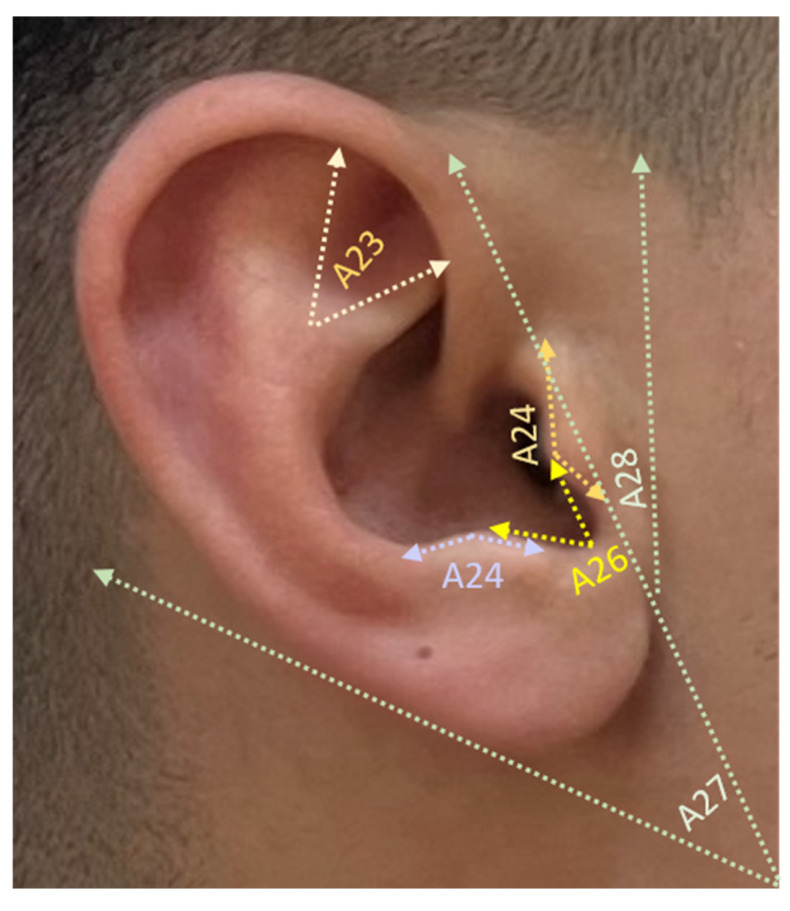
Angular measurements of the auricle (^0^) (Parameters of A23–28). A23. Triangular fossa angle; A24. Antitragal angle; A25. Tragal angle; A26. Intertragic notch angle; A27. Lobular angle; A28. Angle between the preauricular line and the vertical plane.

Digital images were transferred to a computer and analyzed using ImageJ software Version 1.54 (National Institutes of Health, Bethesda, MD, USA), an open-source image analysis platform. All measurements were performed by the same investigator to ensure intra-observer consistency. Auricular morphometry included a comprehensive set of linear and angular variables based on standardized anatomical landmarks. Linear measurements of the auricle (mm) corresponding to parameters A1–A12 are illustrated in Figure 1, parameters A13–A22 are illustrated in Figure 2, and angular measurements (°) corresponding to parameters A23–A28 are illustrated in Figure 3.

Intra-observer reliability assessment

To assess intra-observer reliability, all auricular measurements were repeated three times by the same investigator using the same digital image analysis procedure. Reliability was evaluated using the intraclass correlation coefficient (ICC) based on a two-way mixed-effects absolute-agreement model. Single-measure and average-measure ICC values with 95% confidence intervals were reported.

Statistical Analysis

Statistical analyses were performed using a structured analytical workflow involving IBM SPSS Statistics version 29.0.2 (IBM Corp., Armonk, NY, USA), R version 4.5.3 (R Foundation for Statistical Computing, Vienna, Austria), and MedCalc Statistical Software version 23.0.8 (MedCalc Software Ltd, Ostend, Belgium). Descriptive statistics and initial sex-based comparisons of auricular morphometric parameters were performed using IBM SPSS. Continuous variables were summarized as mean ± standard deviation. Between-sex comparisons were performed using independent-samples *t*-tests, and the magnitude of sexual dimorphism was quantified using Cohen’s d with 95% confidence intervals. Statistical significance was set at *p* < 0.05.

Predictive modeling was performed in R using RStudio 2026.01.2 + 418 (Posit, Boston, MA, USA) as the development environment. Binary logistic regression was used to model female sex. Candidate predictors included age and 28 auricular anthropometric measurements. Because the number of candidate predictors was relatively high in relation to the sample size, and because several auricular measurements were expected to be anatomically correlated, multicollinearity was first screened using tolerance and variance inflation factor (VIF) values. Penalized logistic regression using the least absolute shrinkage and selection operator (LASSO) was then applied for variable selection and shrinkage. Continuous predictors were standardized internally during penalized modeling. Among the penalized solutions, a parsimonious 5-predictor model was selected as the primary model based on the balance between cross-validated discrimination, interpretability, parsimony, and numerical stability. The selected predictors were then refitted in a standard binary logistic regression model to obtain regression coefficients, odds ratios (ORs), and 95% confidence intervals. The glmnet, pscl, ResourceSelection, and car packages were used for penalized regression, pseudo-R^2^ estimation, goodness-of-fit testing, and collinearity diagnostics, respectively.

Receiver operating characteristic (ROC) curve analysis and diagnostic performance evaluation were performed using MedCalc^®^ Statistical Software version 22.023 (MedCalc Software Ltd., Ostend, Belgium). For ROC analysis, predicted probabilities obtained from the final 5-predictor logistic regression model were used as the test variable, and biological sex was used as the binary outcome. The area under the ROC curve (AUC) was calculated to quantify discriminative performance. The optimal decision threshold was determined using the Youden index, and sensitivity, specificity, positive predictive value, and negative predictive value were calculated at this threshold.

Additional model performance indices, including the likelihood-ratio test, pseudo-R^2^ measures, Akaike’s information criterion (AIC), the Hosmer–Lemeshow goodness-of-fit test, the Brier score, and variance inflation factor (VIF) values, were calculated in R. To assess potential model optimism and overfitting, internal validation of the final 5-predictor logistic regression model was performed using 1000 bootstrap resamples. In each bootstrap sample, the same 5-predictor logistic regression model was refitted, and its performance was evaluated both in the bootstrap sample and in the original dataset. The average difference between bootstrap-sample performance and original-dataset performance was used to estimate optimism, and optimism-corrected AUC and Brier score values were calculated. Model calibration was assessed using the Hosmer–Lemeshow goodness-of-fit test, the Brier score, and a calibration plot comparing observed and predicted probabilities from the final 5-predictor logistic regression model.

A more complex 8-predictor model was additionally examined as a sensitivity analysis. However, because this model generated a warning indicating numerically complete or near-complete separation and showed higher collinearity, it was not selected as the primary model despite its higher apparent AUC.

## 3. Results

The descriptive statistics and comparative analysis of the 28 auricular morphometric parameters are presented in Table 1. Intra-observer reliability was good to excellent. In the reliability evaluation, average-measure ICC values ranged from 0.855 to 0.993 using a two-way mixed-effects absolute-agreement model. Since the mean of the three repeated measurements was used in the final analyses, the average-measure ICC was considered the primary reliability estimate. A comprehensive evaluation revealed that the majority of linear and angular measurements exhibited statistically significant sexual dimorphism. Linear measurements and magnitude of difference statistical analysis indicated that almost all linear dimensions (A1–A22) were significantly larger in males compared to females (*p* < 0.001). The most substantial differences, as indicated by Cohen’s d effect sizes, were observed in auricular width (A2) (d = 2.10; 95% CI: 1.66–2.54), auricular width at the level of tragus (A3) (d = 1.97; 95% CI: 1.53–2.41), and auricular height (A1) (d = 1.49; 95% CI: 1.08–1.89). These findings demonstrate a “very large” effect size, suggesting these parameters are robust indicators for sex determination. In contrast to the linear dimensions, angular measurements demonstrated more variable and generally less pronounced sexual dimorphism. While the tragal angle (A25) and triangular fossa angle (A23) showed significant differences (*p* = 0.012 and *p* = 0.041, respectively), the magnitude of these differences was relatively low. Furthermore, no statistically significant differences were observed in the antitragal angle (A24), intertragic notch angle (A26), and lobular angle (A27) (*p* > 0.05), indicating these specific features may not be reliable for sex differentiation in this population. A notable finding was observed regarding the angle between the preauricular line and the vertical plane (A28). Unlike most other parameters, this angle was significantly greater in females (19.18 ± 6.32) than in males (13.28 ± 3.58), yielding a strong negative effect size −1.14 (−1.53–−0.76). This inverse relationship highlights the parameter’s unique contribution to the predictive model.

**Figure 4:**
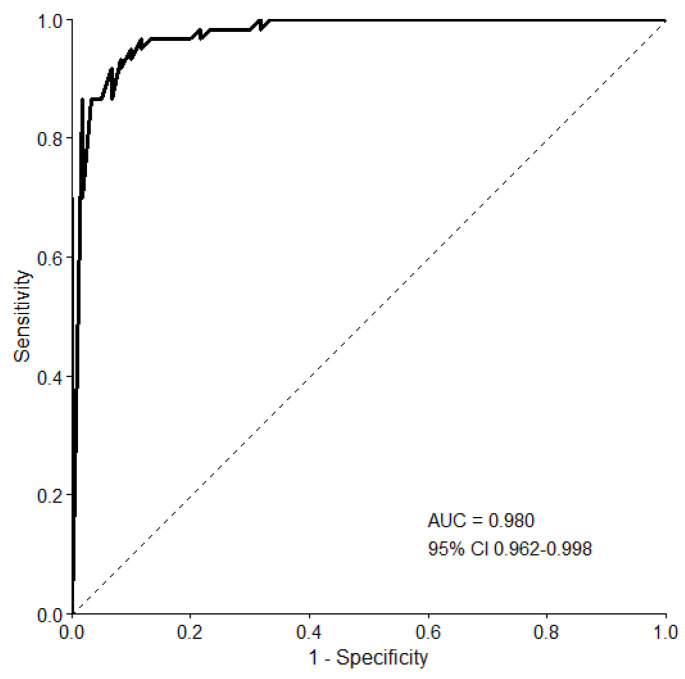
Receiver operating characteristic curves for the primary 5-predictor model for predicting female sex.

**Figure 5:**
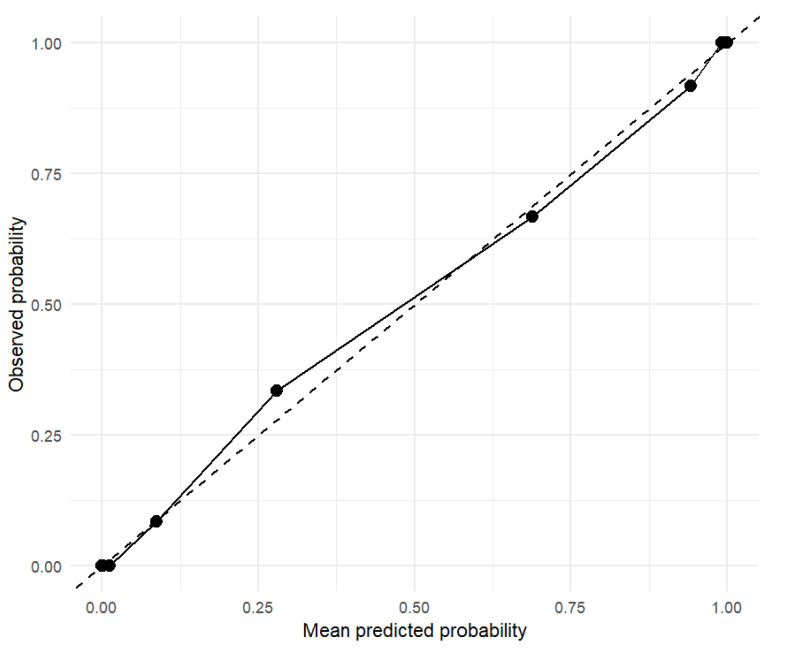
Calibration plot of the final 5-predictor logistic regression model for predicting female sex.

Among the more parsimonious penalized solutions, a 5-predictor model was selected as the primary model because it provided strong cross-validated discrimination while offering greater parsimony, interpretability, and numerical stability than more complex alternatives (Table 2). This model included A2, A3, A5, A10, and A28 and showed a cross-validated AUC of 0.949. After refitting these predictors in a standard binary logistic regression model, overall model fit was strong, with a likelihood-ratio χ^2^ of 123.19 (df = 5, *p* < 0.001). Apparent discrimination was high (AUC = 0.980, 95% CI 0.962–0.998), with an AIC of 55.16 (Figure 4). The Hosmer–Lemeshow test did not indicate lack of fit (*p* = 0.9997), and the Brier score was 0.0567. Graphical calibration assessment showed generally acceptable agreement between predicted and observed probabilities in the final 5-predictor model, although interpretation should be cautious given the modest sample size (Figure 5). In the refitted model, A2 (OR = 0.73, 95% CI 0.54–0.94, *p* = 0.026), A10 (OR = 0.36, 95% CI 0.15–0.70, *p* = 0.008), and A28 (OR = 1.51, 95% CI 1.21–2.08, *p* = 0.002) remained statistically significant. A3 and A5 were retained because they were selected during the penalized modeling stage and contributed to the overall multivariable predictive structure, although they did not remain individually significant after unpenalized refitting. Given the anatomical correlation among auricular width-related measurements, their nonsignificant individual coefficients were interpreted as reflecting shared variance within the model rather than the absence of predictive contribution. Therefore, they were not removed solely on the basis of post-selection *p*-values. Variance inflation factors ranged from 1.26 to 2.10 in the final 5-predictor model, indicating no substantial residual multicollinearity.

At the optimal probability threshold determined by the Youden index, the final 5-predictor model demonstrated strong diagnostic classification performance for predicting female sex (Table 3). The optimal decision threshold was 0.304, with a Youden index of 0.850. At this threshold, sensitivity was 96.7%, specificity was 88.3%, positive predictive value was 89.2%, and negative predictive value was 96.4%. The corresponding confusion matrix showed 58 true-positive, 53 true-negative, 7 false-positive, and 2 false-negative classifications.

To evaluate potential model optimism, internal validation of the final 5-predictor logistic regression model was performed using 1000 bootstrap resamples. The mean bootstrap optimism for AUC was 0.009, resulting in an optimism-corrected AUC of 0.971. The optimism-corrected Brier score was 0.0746. These findings indicate that the model retained strong discriminative performance after internal validation, although a small degree of optimism was observed. This internal validation approach was particularly important because 28 candidate predictors were initially considered in a modest sample of 120 participants.

In a sensitivity analysis, a more complex 8-predictor model yielded higher apparent performance metrics (AUC = 0.994; AIC = 43.32). However, this model generated the warning “fitted probabilities numerically 0 or 1 occurred” and showed higher collinearity, particularly for A28 (VIF = 8.35). This warning indicates numerically complete or near-complete separation, meaning that certain combinations of predictors almost perfectly classified the outcome within the study sample. Under such conditions, maximum-likelihood coefficient estimates may become unstable, predicted probabilities may be overly extreme, and apparent discrimination may be overestimated. Therefore, despite its higher apparent AUC, the 8-predictor model was considered less robust and less generalizable than the primary 5-predictor model. For this reason, it was retained only as a sensitivity analysis and was not selected as the primary model.

## 4. Discussion

The present study demonstrates that auricular morphometry is a highly effective tool for sex estimation, with linear dimensions providing significantly greater predictive power compared to angular measurements. Our findings highlight a distinct pattern of sexual dimorphism within the studied population, which both aligns with and diverges from international literature in terms of specific mean values and the most dimorphic parameters. The most consistent finding across all compared studies is that male auricular dimensions are significantly larger than those of females (*p* < 0.001 for all linear parameters).

Sexual dimorphism in external ear dimensions has been reported in previous studies. Previous studies have consistently demonstrated significant differences in auricular linear measurements between males and females, with males generally exhibiting larger values [18,19].

The human external ear, or pinna, serves as the most distinctive landmark of the craniofacial region, offering vital clues for age and sex estimation [20,21,22,23].

In line with this, multiple population-specific studies across various age groups have consistently demonstrated that the mean length of the external ear in males is significantly greater than that observed in females.

In our study, the auricular height (A1) was measured at 83.58 ± 9.49 and 71.09 ± 7.10 mm for males and females, respectively. These values are notably higher than those reported by [17] in a similar Turkish cohort of university students, where the auricular length (PAL) of the right side was 61.79 ± 5.00 mm in males and 57.12 ± 3.80 mm in females. In a study [24] the authors used image-based analysis on a Turkish sample to find an ear length of 61.70 ± 4.29 mm for males and 58.17 ± 4.26 mm for females. This variation may reflect population-specific anthropometric variability, differences in sample composition, and methodological heterogeneity among studies. Furthermore, [25] demonstrated the impact of ethnicity on these dimensions; they reported ear lengths of 65.6 ± 4.0 mm in a Malaysian male population and 62.3 ± 4.2 mm in an Indian male population, both of which remain significantly lower than the A1 values observed in our study.

Similarly, our values for auricular width (A2) (Male: 48.01 ± 5.48 mm, Female: 37.71 ± 4.23 mm) exceeded the measurements reported [26] in a Sudanese population (Male: 34.22 ± 2.75 mm and Female: 32.24 ± 2.31 mm). The significant difference in width between our results and the Sudanese data underscores the necessity of population-specific standards for forensic identification. In terms of the magnitude of sexual dimorphism, our study identified auricular width (A2) as the most dimorphic parameter with a Cohen’s d of 2.10, followed by width at the level of the tragus (A3, d = 1.97). This contrasts with the findings of [9], who observed that in a Nepalese population, auricular height was the most reliable indicator for sex determination. Furthermore, [27] reported that while both height and width were dimorphic in an Indian sample (Right Ear Height, Male: 63.7 ±0.65 mm; Female: 61.1 ± 0.47 mm), height generally provided higher classification accuracy [26]. The high effect sizes observed for width in our study suggest that, for the Turkish population, horizontal dimensions may be more critical for sex estimation than previously emphasized in other ethnic groups.

Our analysis of angular measurements (A23–A27) revealed a more complex and less dimorphic landscape. The triangular fossa angle (A23) was significantly wider in females (51.49 ± 11.070) than in males (47.32 ± 8.240, *p* = 0.041). In contrast, parameters such as the lobular angle (A27) did not show significant differences (*p* = 0.330). This lack of dimorphism in certain ear sub-regions is echoed by [14], who noted that lobular width (Male: 21.18 ± 3.90 mm, Female:22.17 ± 3.5 mm) and height (22.16 ± 7.03 mm, 22.88 ± 9.00 mm; male and female, respectively) did not consistently differ between sexes in their clinical sample from Nepal. This suggests that while the overall frame of the ear is larger in males, specific structural angles may follow different biological or developmental patterns.

The robustness of our logistic regression model—incorporating A2, A10, and A28—is supported by the high accuracy rates found in the literature. In a study [26] the authors achieved an overall accuracy ranging between 60.5% and 72% using anthropometric variables of the ear.

In a study [15] the authors highlight the superior precision of automated 3D algorithms over manual methods (as cited in their technical validation of CT-based measurements), our study confirms that traditional image-based morphometry remains a powerful and accessible tool in forensic contexts where advanced 3D scanning may not be available.

When interpreting comparisons with previous studies, methodological differences should be carefully considered. The studies cited in the literature employed diverse measurement techniques, including direct caliper-based anthropometry, two-dimensional photographic analysis, and three-dimensional imaging systems. These approaches differ in terms of landmark identification, image calibration procedures, measurement precision, and susceptibility to observer-related error. Therefore, variations observed between studies may not solely reflect population-specific biological differences but may also be influenced by methodological heterogeneity. Consequently, direct comparisons of absolute auricular dimensions and diagnostic performance metrics across studies should be interpreted with caution. Nevertheless, despite these methodological differences, the overall pattern of sexual dimorphism observed in the present study remains largely consistent with findings reported in the international literature.

Another methodological consideration is that only the right auricle was analyzed in the present study. This approach was adopted to ensure measurement standardization and consistency across participants. Previous studies have reported that auricles generally demonstrate a high degree of bilateral symmetry, with most linear, area, and ratio measurements showing good agreement between the right and left sides, although certain angular measurements may exhibit asymmetry [28,29,30].

This study provides a comprehensive analysis of auricular morphometry and its efficacy in sex estimation within a Turkish population. The findings confirm that the external ear exhibits significant sexual dimorphism, particularly in its linear dimensions. Among the 28 parameters evaluated, auricular width (A2) and auricular width at the level of the tragus (A3) emerged as the most robust indicators of sex, demonstrating “very large” effect sizes that surpass many traditional anthropometric markers.

The development of a parsimonious 5-predictor logistic regression model (incorporating A2, A3, A5, A10, and A28) proved to be highly effective, achieving an exceptional discriminative performance with an AUC of 0.980. A key finding of this study is the unique contribution of the angle between the preauricular line and the vertical plane (A28), which, unlike most linear measurements, was significantly larger in females, providing a critical inverse relationship that enhances the model’s predictive accuracy.

The selected predictors correspond to anatomically interpretable auricular measurements, including auricular width (A2), auricular width at the level of the tragus (A3), distance between the crus helicis and conchal margin (A5), tragal width (A10), and the angle between the preauricular line and the vertical plane (A28). Together, these variables represent complementary dimensions of auricular morphology, including linear size, contour-related structure, and angular orientation. Their forensic relevance lies in the fact that such measurements can be obtained from standardized auricular photographs and may contribute to sex estimation when conventional biological identifiers are unavailable, degraded, or incomplete. Therefore, these predictors should be interpreted not merely as statistical variables, but as components of a multivariable morphometric pattern with potential applicability in forensic sex estimation. Comparative analysis with international literature underscores the high degree of biological variation across different ethnic groups. The significantly higher mean values for auricular height and width observed in this study compared to Sudanese, Indian, and even other Turkish cohorts highlight the indispensable need for population-specific standards in forensic anthropology. Relying on universal averages may lead to significant misclassification in forensic identification and clinical diagnosis.

Our study demonstrated that the linear dimensions of the external ear exhibit pronounced sexual dimorphism in biological sex estimation, with male participants displaying significantly larger auricular parameters than females. These findings are in strong agreement with the studies of [30,31], who similarly reported that absolute linear anthropometric measurements, such as ear length and width, possess substantially greater discriminatory power for sex determination than proportional indices [30,31]. Furthermore, the studies [25,32] further support these observations, emphasizing that normal auricular morphometric measurements constitute practical, reliable, and cost-effective parameters for forensic sex estimation [25,32]. In the present study, the comprehensive evaluation of 22 linear auricular parameters using ImageJ software demonstrated that linear morphometry provides a robust and holistic framework for logistic regression-based sex prediction models.

While conventional forensic anthropological research has largely focused on isolated measurements of the auricle, topographical analyses have demonstrated that the spatial positioning of the ear relative to the anterior and posterior facial midlines may provide valuable secondary biometric markers for individual identification [33]. The logistic regression modeling approach applied in the present study identified the most informative variables within a multivariable morphometric dataset by integrating selected linear dimensions and angular relationships. The high classification accuracy achieved by the ROC-validated model suggests that these auricular parameters may serve as preliminary morphometric references for forensic medicine and forensic clinical anatomy. However, although the findings may inform future automated biometric studies, the present study did not implement a machine learning or artificial intelligence-based classification framework. Therefore, the applicability of these parameters to AI-assisted biometric recognition systems should be evaluated in future studies using dedicated machine learning algorithms and independent validation datasets.

## 5. Limitations

Several limitations of this study should be acknowledged. First, the study was conducted in a single-center Turkish adult sample with a relatively limited sample size. Participants were recruited using a non-probability convenience sampling approach rather than probability-based population sampling. Therefore, potential selection bias cannot be fully excluded, and the sample may not fully represent the broader Turkish population or other ethnic and geographical groups.

Second, auricular measurements were obtained using two-dimensional photographic analysis rather than advanced three-dimensional imaging systems, which may affect the precision of certain morphometric parameters.

Third, the relatively high number of initial candidate predictors compared with the sample size represents a potential source of model instability. Although 28 auricular measurements were evaluated in a cohort of 120 participants, these predictors were not entered simultaneously into a conventional full logistic regression model. Instead, LASSO penalized logistic regression was used for variable selection and shrinkage, and the final model was restricted to five predictors to improve parsimony and numerical stability. Internal bootstrap validation showed that the model retained strong discriminative performance after optimism correction. Nevertheless, given the modest sample size and the anatomical correlation among auricular measurements, some degree of model instability cannot be fully excluded. Accordingly, the excellent diagnostic performance observed in this cohort may partly reflect sample-specific morphometric characteristics, and the generalizability of the proposed logistic regression model to broader populations should therefore be interpreted with caution. The sensitivity analysis further suggested that increasing model complexity may improve apparent discrimination but at the cost of numerical stability, as evidenced by near-complete separation and increased collinearity in the 8-predictor model.

Fourth, the model was not externally validated in an independent cohort; therefore, the reported performance should be interpreted as internally validated rather than externally generalizable. In addition, although the findings may inform future automated biometric studies, no machine learning or artificial intelligence-based classification framework was implemented in the present study.

Finally, the cross-sectional nature of the study limits the evaluation of age-related longitudinal morphological changes in the auricle. Future studies with larger, independent, population-based, and ethnically diverse samples are required to confirm the transportability and forensic applicability of the proposed model.

## 6. Conclusions

In conclusion, this study provides an internally validated auricular morphometric model for biological sex estimation in a Turkish adult sample. The findings confirm that selected auricular parameters, particularly linear dimensions and the angle between the preauricular line and the vertical plane, show substantial discriminative value for sex estimation. The proposed 5-predictor logistic regression model demonstrated high discriminative performance and may provide a preliminary reference for future forensic identification studies when auricular soft-tissue morphology is available. Furthermore, these findings may have potential relevance for future biometric recognition research. However, because no artificial intelligence-based classification framework was implemented in this study, the applicability of these parameters to AI-assisted biometric systems should be confirmed in future studies using dedicated machine learning approaches and independent validation datasets. Future research should aim to expand these findings across broader age groups, larger independent cohorts, and diverse geographical regions to further refine the understanding of auricular biological variation.

## Figures and Tables

**Table 1:** Descriptive statistics and sexual dimorphism of auricular measurements, including significance levels and effect sizes (Cohen’s d).

Parameters	Male Mean ± SD	Female Mean ± SD	*p*	Cohen’s d (95% CI)
A1—Auricular height	83.58 ± 9.49	71.09 ± 7.10	<0.001	1.49 (1.08 to 1.89)
A2—Auricular width	48.01 ± 5.48	37.71 ± 4.23	<0.001	2.10 (1.66 to 2.54)
A3—Auricular width at the level of tragus	39.91 ± 5.04	30.97 ± 3.92	<0.001	1.97 (1.53 to 2.41)
A4—Lobular width	26.01 ± 3.85	22.21 ± 3.75	<0.001	0.99 (0.61 to 1.37)
A5—Distance between crus helicis and conchal margin	22.37 ± 3.50	17.46 ± 2.82	<0.001	1.54 (1.13 to 1.94)
A6—Maximum distance between tragus and helix	52.16 ± 6.60	43.10 ± 5.31	<0.001	1.51 (1.10 to 1.91)
A7—Maximum distance between tragus and lobule	33.31 ± 5.40	28.86 ± 3.60	<0.001	0.96 (0.58 to 1.34)
A8—Upper tragal length	9.74 ± 2.36	8.08 ± 1.75	<0.001	0.79 (0.42 to 1.16)
A9—Lower tragal length	9.39 ± 1.82	7.65 ± 1.57	<0.001	1.02 (0.63 to 1.40)
A10—Tragal width	6.20 ± 1.42	4.94 ± 1.17	<0.001	0.96 (0.58 to 1.34)
A11—Tragal length	18.82 ± 3.01	15.81 ± 2.47	<0.001	1.09 (0.70 to 1.47)
A12—Lobular length	20.70 ± 4.01	17.99 ± 2.77	<0.001	0.78 (0.41 to 1.15)
A13—Helical width	4.71 ± 1.41	3.47 ± 0.82	<0.001	1.07 (0.68 to 1.45)
A14—Scapha width	4.41 ± 1.45	3.27 ± 1.02	<0.001	0.90 (0.52 to 1.27)
A15—Antihelical width	5.63 ± 1.51	4.30 ± 1.29	<0.001	0.94 (0.56 to 1.32)
A16—Superior antihelical crus length	16.23 ± 3.95	13.71 ± 2.95	<0.001	0.72 (0.35 to 1.08)
A17—Inferior antihelical crus length	15.23 ± 3.07	13.41 ± 2.24	<0.001	0.67 (0.30 to 1.04)
A18—Distance between crus origin and antitragus	29.90 ± 6.63	25.99 ± 3.53	<0.001	0.94 (0.56 to 1.32)
A19—Conchal length	41.42 ± 4.83	36.28 ± 4.57	<0.001	1.09 (0.70 to 1.47)
A20—Conchal width	23.60 ± 3.65	19.04 ± 3.00	<0.001	1.36 (0.96 to 1.75)
A21—Tragus–antitragus distance	11.12 ± 2.50	9.07 ± 2.02	<0.001	0.90 (0.52 to 1.27)
A22—Intertragic notch length	10.35 ± 1.61	8.64 ± 1.84	<0.001	0.98 (0.60 to 1.36)
A23—Triangular fossa angle	47.32 ± 8.24	51.49 ± 11.07	0.041	−0.42 (−0.78 to −0.06)
A24—Antitragal angle	134.63 ± 11.45	131.64 ± 13.09	0.186	0.24 (−0.11 to 0.60)
A25—Tragal angle	133.64 ± 12.31	128.00 ± 11.99	0.012	0.46 (0.10 to 0.82)
A26—Intertragic notch angle	51.01 ± 11.19	49.70 ± 12.19	0.539	0.11 (−0.24 to 0.47)
A27—Lobular angle	52.70 ± 7.58	51.18 ± 9.35	0.330	0.17 (−0.18 to 0.53)
A28—Angle between preauricular line and vertical plane	13.28 ± 3.58	19.18 ± 6.32	<0.001	−1.14 (−1.53 to −0.76)

**Table 2:** Binary logistic regression model predicting female sex.

Predictor	B	SE	z	*p*	OR	95% CI for OR
A2—Auricular width	−0.309	0.139	−2.223	0.026	0.73	0.54, 0.94
A3—Auricular width at the level of tragus	−0.208	0.127	−1.635	0.102	0.81	0.61, 1.03
A5—Distance between crus helicis and conchal margin	−0.275	0.185	−1.489	0.137	0.76	0.51, 1.07
A10—Tragal width	−1.029	0.387	−2.660	0.008	0.36	0.15, 0.70
A28—Angle between preauricular line and vertical plane	0.413	0.135	3.070	0.002	1.51	1.21, 2.08

Note. The outcome was female sex. Predictors were first selected using LASSO logistic regression and then refitted in a standard binary logistic regression model. Model fit was strong, LR χ^2^(5) = 123.19, *p* < 0.001; AIC = 55.16; McFadden R^2^ = 0.741; Nagelkerke/Cragg-Uhler pseudo-R^2^ = 0.856. Apparent discrimination was high, AUC = 0.980, 95% CI [0.962, 0.998], whereas the cross-validated AUC from the penalized selection stage was 0.949. The Hosmer–Lemeshow test did not indicate lack of fit, χ^2^(8) = 0.62, *p* = 0.999. The Brier score was 0.0567. VIF values ranged from 1.26 to 2.10, indicating no substantial residual multicollinearity.

**Table 3:** Diagnostic performance of the final 5-predictor model at the optimal ROC threshold.

Metric	Value
AUC (95% CI for AUC)	0.980 (0.962–0.998)
Optimal probability threshold	0.304
Youden index	0.850
Sensitivity	96.7%
Specificity	88.3%
Positive predictive value	89.2%
Negative predictive value	96.4%
True positives	58
True negatives	53
False positives	7
False negatives	2
Optimism-corrected AUC	0.971
Optimism-corrected Brier score	0.0746

Note. Positive classification refers to female sex. The optimal probability threshold was determined using the Youden index. Positive and negative predictive values were calculated according to the study sample distribution.

## Data Availability

The original contributions presented in this study are included in the article. Further inquiries can be directed to the corresponding author.
